# The Proteasome Inhibitor, MG132, Attenuates Diabetic Nephropathy by Inhibiting SnoN Degradation *In Vivo* and *In Vitro*

**DOI:** 10.1155/2014/684765

**Published:** 2014-06-09

**Authors:** Wei Huang, Chen Yang, Qinling Nan, Chenlin Gao, Hong Feng, Fang Gou, Guo Chen, Zhihong Zhang, Pijun Yan, Juan Peng, Yong Xu

**Affiliations:** ^1^Department of Endocrinology, Affiliated Hospital of Luzhou Medical College, Luzhou, Sichuan 646000, China; ^2^Daping Hospital of Third Military Medical University, Chongqing 400042, China

## Abstract

Transforming growth factor-**β** (TGF-**β**) has been shown to be involved in diabetic nephropathy (DN). The SnoN protein can regulate TGF-**β** signaling through interaction with Smad proteins. Recent studies have shown that SnoN is mainly degraded by the ubiquitin-proteasome pathway. However, the role of SnoN in the regulation of TGF-**β**/Smad signaling in DN is still unclear. In this study, diabetic rats were randomly divided into a diabetic control group (DC group) and a proteasome inhibitor (MG132) diabetes therapy group (DT group). Kidney damage parameters and the expression of SnoN, Smurf2, and TGF-**β** were observed. Simultaneously, we cultured rat glomerular mesangial cells (GMCs) stimulated with high glucose, and SnoN and Arkadia expression were measured. Results demonstrated that 24-hour urine protein, ACR, BUN, and the expression of Smurf2 and TGF-**β** were significantly increased (*P* < 0.05), whereas SnoN was significantly decreased in the DC group (*P* < 0.05). However, these changes diminished after treatment with MG132. SnoN expression in GMCs decreased significantly (*P* < 0.05), but Arkadia expression gradually increased due to high glucose stimulation (*P* < 0.05), which could be almost completely reversed by MG132 (*P* < 0.05). The present results support the hypothesis that MG132 may alleviate kidney damage by inhibiting SnoN degradation and TGF-**β** activation, suggesting that the ubiquitin-proteasome pathway may become a new therapeutic target for DN.

## 1. Introduction

Diabetic nephropathy (DN) is one of the most prevalent and serious microvascular complications of diabetes mellitus (DM) [1]. Early pathological characteristics are basement membrane thickening, increased mesangial matrix production, and extracellular matrix accumulation, with subsequent development of glomerulosclerosis and tubulointerstitial fibrosis, eventually leading to irreversible renal damage [2–4]. Currently, the pathogenesis of DN remains unclear and treatments such as strict glucose and blood pressure control are limited in their effectiveness [5]. Further investigations into molecular mechanisms are required, in order to develop new therapeutical approaches for DN.

As a key mediator of fibrogenesis, transforming growth factor-*β* (TGF-*β*) plays a critical role in the development of DN [6]. Many fibrogenic cytokines, such as advanced glycation end products (AGEs), due to hyperglycemia, may activate TGF-*β* signaling by a Smad-dependent pathway, resulting in fibrosis [7].

The transcriptional coregulator, SnoN, is a critical and versatile regulator of TGF-*β*-induced transcription and responses. SnoN controls TGF-*β*-mediated responses by acting as a transcriptional corepressor or transcriptional coactivator [8]. SnoN associates with Smad2/3 and Smad4 and is recruited to TGF-*β* responsive genes, thus influencing their transcription [9]. Remarkably, as well as inducing SnoN degradation, TGF-*β* stimulates SnoN transcription; once expressed, SnoN acts as a negative feedback inhibitor of TGF-*β* signaling. When overexpressed, SnoN inhibits transcription of genes regulated by the TGF-*β*/Smad signaling pathway [9, 10]. To counteract SnoN inhibition of transcription, TGF-*β* signaling induces the degradation of SnoN by the ubiquitin-proteasome pathway (UPP) [11, 12]. SnoN expression is altered under many pathological conditions including wound healing, liver regeneration, and obstructive nephropathy [13].

Ubiquitin is well known for its function in targeting proteins for degradation by the 26S proteasome, which is important for the removal of abnormal and damaged proteins and many regulated processes. Ubiquitin ligases, such as Smurf2 and Arkadia, mediate the ability of TGF-*β* to induce ubiquitination and consequent degradation of SnoN [14]. Smad ubiquitin regulatory factor 2 (Smurf2) is an E3 ubiquitin ligase that regulates transforming growth factor-*β* (TGF-*β*)/Smad signaling and is implicated in a wide variety of cellular responses [15]. Arkadia is a nuclear protein with 989 amino acid residues, with a characteristic C-terminal RING domain [16]. Arkadia appears to effectively enhance TGF-*β* signaling through simultaneous downregulation of two distinct types of negative regulators, namely, Smad7 and SnoN, which are critical substrates of Arkadia and may play an important role in determining the intensity of TGF-*β* family signaling in target cells [17].

Previous studies have demonstrated that AGEs, formed as a result of hyperglycemia, can activate TGF-*β* signaling in DN. SnoN, as a negative regulator of TGF-*β* signaling, can be degraded by the UPP. However, whether ubiquitin degradation of SnoN, by TGF-*β* signaling, is involved in the development of DN still remains to be elucidated. Here, we established a rat model of DN by using STZ and selected MG132 as the specific ubiquitin-proteasome inhibitor to block the UPP, in order to explore the relationship between the UPP and the TGF-*β*/Smad signaling pathway* in vivo*. We also investigated whether SnoN is degraded and if UPP is activated in cultured rat glomerular mesangial cells (GMCs) stimulated by high glucose* in vitro*.

## 2. Materials and Methods

### 2.1. Establishing the Animal Model

Male Wistar rats weighing 200 g were purchased from the Biotechnology Corporation of Teng Xing (Chongqing, China). Rats were brought into a special room with a stable ambient temperature of 18°C–22°C and housed in wire cages with free access to a standard diet and tap water. Blood glucose levels of all rats were measured prior to the start of the experiment.

The rats were randomly allocated into two groups: a control group (NC group, *n* = 20) and an experimental group. Rats in the experimental group were rendered diabetic by intraperitoneal injection of Streptozocin (STZ, Sigma, USA), at a dose of 60 mg/kg. STZ was dissolved in 0.1 M citrate buffer at pH 4.5. Meanwhile, rats in the NC group received an intraperitoneal injection of the same volume of citrate buffer. After 3 days following the STZ injection, fasting glycemic measurements were performed in blood samples from tail veins, and blood glucose levels of ≥16.7 mmol/L lasting 3 days were confirmed as being “diabetic.” Diabetic rats that presented mild microalbuminuria (an early sign of DN) were all included in the study and were further randomly divided into two groups: a diabetic control group (DC group, *n* = 20) and a diabetes therapy group (DT group, *n* = 20), treated with MG132 (0.05 mg/kg daily, CALBIOCHEM, USA). Meanwhile, the NC and DC groups received daily injections of equivalent volumes of citrate buffer.

### 2.2. Cell Culture

Rat GMCs (HBZY-1) were purchased from the Preservation Center at Wuhan University and maintained in low-glucose Dulbecco's modified eagle medium (DMEM) with 10% fetal bovine serum (Hyclone) at 37°C and 5% CO_2_. GMCs were used for all experiments and randomly divided into the following five groups: normal control group (NC group, with medium containing 5.6 mmol/L glucose), 20 mmol/L glucose group (20 M group, with medium containing 20 mmol/L glucose), 30 mmol/L glucose group (30 M group, with medium containing 30 mmol/L glucose), osmotic pressure control group (OP group, with medium containing 5.6 mmol/L glucose + 24.6 mmol/L mannitol), and MG132 therapy group (MT group, with medium containing 30 mmol/L glucose + 0.5 *μ*mol/L MG132) to block the UPP. Cells in each group were cultured for 12 h, 24 h, and 48 h to detect SnoN and Arkadia expression by Western blotting, RT-PCR, and immunofluorescence.

### 2.3. Sample Collection and Body Weight: Biochemical Measurements

All rats were weighed and 24-hour urinary microalbumin (mAlb) was collected every day. Urinary protein and urinary creatinine concentrations were measured according to the manufacturers' procedures described in the kits, and urine albumin-creatinine ratios (ACR) were calculated. After 6 or 8 weeks of MG132 (DT group) or citrate buffer (NC and DC group) injections, all rats were sacrificed and heart blood was collected to measure BUN levels and fasting blood glucose (FBG) levels, using an automatic biochemistry analyzer. Both kidneys were weighed and cut along the coronal plane; upper poles of the right kidneys were used for pathology, and the left renal tissues were preserved at −80°C until required for Western blot analysis and RT-PCR.

### 2.4. Morphological Analysis: Mesangial Expansion and Collagen

After 6 or 8 weeks, the animals were killed and the upper poles of the right kidneys were rapidly removed, fixed in 10% formaldehyde, dehydrated with gradient ethanol, embedded in paraffin, and sectioned at 4 *μ*m thickness. Renal sections were stained with HE and Masson staining. All sections were evaluated under a light microscope. The glomerular cross-sectional area (Ag), mesangial area (Am), and tuft area (At) were measured in 20 glomerular profiles per rat by using Image-Pro Plus 6.0 software. The values of semiquantitative analysis for the positive areas are expressed as the means ± SD from rat at each group. All measurements were done in a masked fashion.

### 2.5. Immunohistochemical Staining

Sections were incubated with the following primary antibodies: SnoN (mouse, 1 : 100 dilution, Santa Cruz, USA) and Smurf2 (rabbit, 1 : 200 dilution, Beijing biosynthesis biotechnology, China) overnight at 4°C. After sections were washed with PBS, they were incubated with horseradish peroxidase-conjugated secondary antibodies (1 : 200 dilution) for 2 h at room temperature. For visualizing the signals, sections were treated with peroxidase substrate DAB (3,3-diaminobenzidine) and counterstained with hematoxylin.

### 2.6. Western Blotting

Renal tissues and rGMCs were homogenized in lysis buffer (Kaiji, Shanghai, China). Proteins were separated by sodium dodecyl sulfate-polyacrylamide gel electrophoresis and transferred to a polyvinylidene difluoride (PVDF) membrane (Millipore). Immunoblot analysis was performed using SnoN antibody (mouse, 1 : 1000; Santa Cruz, USA), TGF-*β* antibody (rabbit, 1 : 1000; CST, USA), *β*-actin antibody (rabbit, 1 : 1000; Abcam, USA), and GAPDH antibody (mouse, 1 : 2000; Beyotime China). Horseradish peroxidase-conjugated secondary antibodies were obtained from the Beyotime Institute of Biotechnology (Shanghai, China). Proteins were detected using the enhanced chemiluminescence system and ECL Hyperfilm (Amersham, England).

### 2.7. RNA Extraction and Reverse-Transcription Polymerase Chain Reaction

Total RNA was extracted from renal tissues and rGMCs using an RNA extraction kit (Tiangen Biotech, Beijing, China). Total RNA was reverse-transcribed (RT) using a Takara RNA PCR kit (Baoshengwu, Dalian, China). cDNA was amplified in a gradient thermal cycler (Eppendorf, Germany) using polymerase chain reaction (PCR) Master Mix (Baoshengwu, Dalian, China). The results were determined using an ultraviolet transilluminator and normalized to glyceraldehyde 3-phosphate dehydrogenase (GAPDH) gene expression. The primer sequences were the following: SnoN (forward, 5′-GAAGAAAAGAAACTGAAGAT-3′, reverse, 5′-CTGGGGTGTAAAAATGAAT-3′) and GAPDH (forward, 5′-CCT CAA GAT TGT CAG CAA T-3′, reverse, 5′-CCA TCC ACA GTC TTC TGA GT-3′).

### 2.8. Immunofluorescence

Mesangial cells were grown on coverslips in 6-well plates. After overnight adherence, cells were treated with media that contained high glucose and MG132 for 24 h. Cells were then fixed in 4% paraformaldehyde (Pierce Biotechnology, Rockford, IL, USA) and blocked with 5% goat serum, followed by incubation with anti-SnoN antibody or anti-Arkadia antibody, overnight at 4°C. After washing, cells were incubated with fluorescein isothiocyanate- (FITC-) conjugated secondary antibodies (BioSynthesis) for 45 min in the dark. Images were taken with a DMIRE2 laser scanning confocal microscope (Leica, Germany).

### 2.9. Statistical Analyses

Each experiment was repeated at least twice. Data are expressed as mean ± standard deviation (SD). Differences were statistically analyzed using one-way analysis of variance (ANOVA), followed by the Least Significant Difference post hoc test for multiple comparisons. A probability value, *P*, of <0.05 was considered significant.

## 3. Results

### 3.1. MG132 Reversed STZ-Induced Changes in 24-Hour Urine Protein and Renal Function of Diabetic Rats

**Figure 1:**
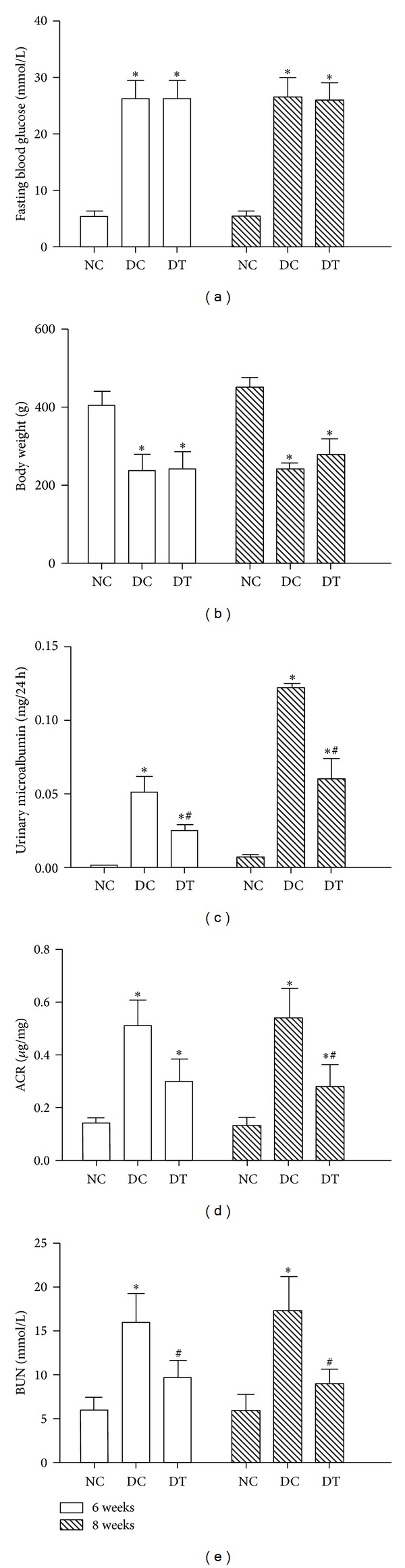
Therapeutic effects of the proteasome inhibitor, MG132, on diabetes-induced general changes and renal function. Diabetic rats were divided into two groups: a diabetic control group (DC group) and a diabetes therapy group (DT group) treated daily with MG132 (0.05 mg/kg). Meanwhile, the NC and DC groups received daily injections of equivalent volumes of citrate buffer. After 6 or 8 weeks, fasting blood glucose (a), body weight (b), 2-hour urine protein (c), ACR (d), and BUN (e) levels were examined before and after treatment with MG132. Data are presented as means ± SD. *P < 0.05 versus NC group; ^#^ *P* < 0.05 versus DC group.

Compared to the NC group (*P* < 0.05), fasting blood glucose (FBG, Figure 1(a)) levels were increased in the DC and DT groups, and the body weight (Figure 1(b)) was significantly decreased (*P* < 0.05). There were, however, no obvious differences in FBG levels and body weight between the DT and DC groups. These data suggest that diabetic rat models were successfully achieved by using STZ and that MG132 was unable to influence STZ-induced changes in FBG and body weight. Conversely, compared to the NC group (*P* < 0.05), 24-hour urine protein (Figure 1(c)), urinary albumin-to-creatinine ratios (ACR, Figure 1(d)), and BUN levels (Figure 1(e)) increased in the DC and DT groups, all important features of DN. Moreover, compared with the DC group (*P* < 0.05), STZ-induced changes were partially reversed by MG132.

### 3.2. MG132 Attenuates STZ-Induced Downregulation of SnoN and Activation of TGF-*β In Vivo*

**Figure 2:**
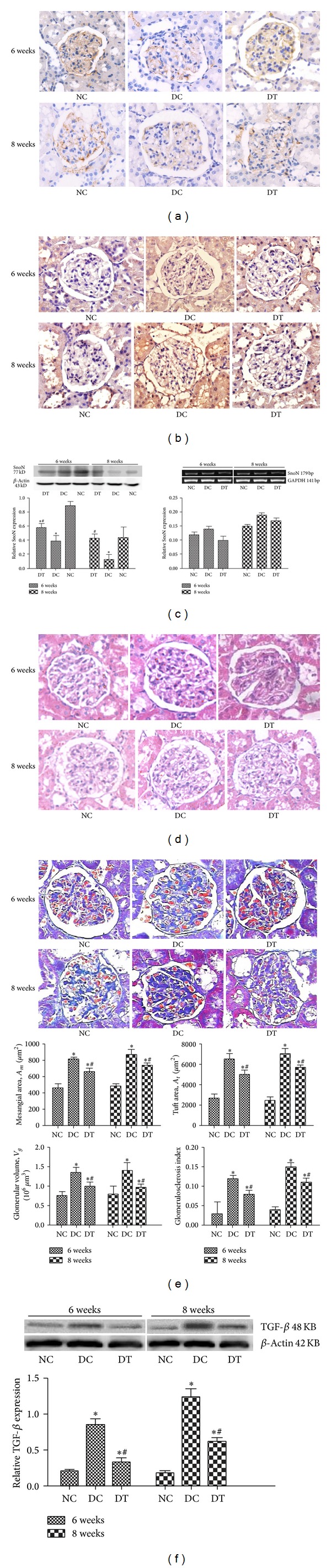
MG132 attenuates STZ-induced downregulation of SnoN and TGF-*β* activation* in vivo*. Representative images of immunohistochemistry staining showing SnoN (a) and Smurf2 (b) expression in renal tissue. SnoN and Smurf2 expression are represented as the positive yellow-brown stained area (200x). SnoN expression in renal tissues was detected by Western blotting and RT-PCR (c): SnoN expression decreased in the DC and DT groups after injection of STZ and MG132 partially reversed SnoN degradation in the DT group; the gray graph confirmed these trends. However, the mRNA levels of SnoN were not statistically different in each group (*P* > 0.05). Morphologic parameters of kidney pathology were examined with HE ((d), 200x) and Masson staining ((e), 200x). The gray graph shows the values of semiquantitative analysis for mesangial area (Am), tuft area (At), glomerular cross-sectional area (Ag), and glomerulosclerosis index. TGF-*β* (f) protein expression in each group by Western blotting and the gray graph show the relative statistical values of TGF-*β* for each group. Data are presented as means ± SD. *P < 0.05 versus NC group; ^#^ *P* < 0.05 versus DC group.

Renal tissue immunohistochemistry (Figure 2(a)) showed that, compared with the NC group, there was a decreased SnoN expression that was particularly evident in the DC group, which was partially reversed by MG132 in the DT group. However, an E3 ubiquitin ligase, Smurf2, express (Figure 2(b)) in the DC group was increased and practically returned to normal in DT group (*P* < 0.05). Renal tissue Western blotting and RT-PCR (Figure 2(c)) demonstrated that the expression of SnoN protein was reduced in the DC and DT groups; however, compared with the DC group (*P* < 0.05), SnoN degradation in the DT group was partially reversed by MG132, although the mRNA levels of SnoN were not statistically different in each group (*P* > 0.05).

To investigate the therapeutic effect of MG132 on the kidney, renal pathology was examined with HE staining (Figure 2(d)). Pathological changes in the kidney of diabetic rats were obvious; the glomerular tuft and mesangial area were increased at 6 or 8 weeks. There was a trend for an increase of glomerular volume in DC group compared with NC group. MG132 treatment ameliorated the increase of both tuft area and mesangial area. Collagen plays a critical structural role in renal fibrosis of DN. Observation with the light microscope, following Masson staining, (Figure 2(e)) demonstrated that accumulation of collagen in the kidney of the DC groups was greater than the NC group in gross appearance; this effect was significantly decreased by MG132 treatment. These experiments and figures showed that induction of DN by STZ was evident in the mesangial area, with deposition of abnormal substances, with the DT group being less pronounced than the DC group.

Consistent with the Masson staining patterns, Western blotting (Figure 2(f)) suggested that TGF-*β* expression, an important factor for regulation of fibrosis, was increased in the DC and DT groups (*P* < 0.01), but expression was significantly decreased in the DT group, due to the action of the proteasome inhibitor, MG132, compared with the DC group (*P* < 0.05).

### 3.3. MG132 Partially Reversed High Glucose-Induced Degradation of SnoN *In Vitro*

**Figure 3:**
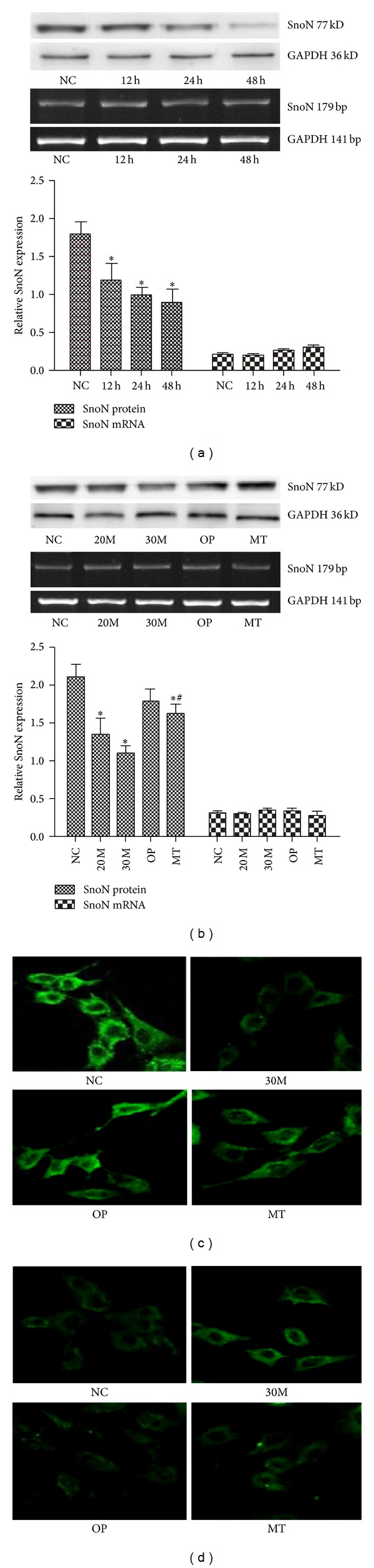
MG132 partially reversed high glucose-induced degradation of SnoN* in vitro*. rGMCs were treated with 30 mmol/L high glucose for 12 h, 24 h, and 48 h (a). Cells were treated with the indicated concentrations of glucose, mannitol, or MG132 for 48 h (b). SnoN expression after high glucose challenge for various times and various glucose concentrations was determined by Western blotting and RT-PCR. The gray graph shows the relative statistical values for SnoN protein and mRNA expression in each group. The data were normalized and are expressed as means ± SD. *P < 0.05 versus NC group; ^#^ *P* < 0.05 versus 30M group; the expressions of SnoN (c) and Arkadia (d) of rGMCs were detected by immunofluorescence and laser scanning confocal microscopy (630x). SnoN and Arkadia were detected in the cytoplasm as green fluorescence.

The relative expression of SnoN (SNON-to-GAPDH protein ratio) decreased as the glucose concentrations and time increased (*P* < 0.05). The most significant changes were observed with 30 mmol/L glucose stimulation after 48 h (Figure 3(a)). SnoN was significantly degraded by different glucose concentrations, especially in the 30 mmol/L glucose group (Figure 3(b)). There was no significant difference between the NC and OP groups with regard to SnoN expression (*P* > 0.05), indicating that the high glucose-induced changes of SnoN were not an osmotic effect. However, the proteasome inhibitor, MG132, partially reversed SnoN degradation. After MG132 treatment, SnoN protein levels in the MT group were partially reversed compared with the 30 mmol/L glucose group. In addition, similar to the experiment* in vivo*, there was no significant difference in each group involving the SnoN mRNA expression. These data suggest that high glucose led to decreased SnoN expression through the UPP.

Immunofluorescence (Figure 3(c)) showed that SnoN is predominantly expressed in the cytoplasm. After 30 mmol/L high glucose intervention, SnoN expression was significantly decreased, but this trend was strongly reversed in the MG132 intervention group, supporting the Western blotting response to high glucose and MG132. Furthermore, Arkadia (Figure 3(d)) was weakly expressed in the NC group but was prominent in the 30 mmol/L high glucose group and did not significantly change as the result of high osmotic pressure after culturing for 24 h, suggesting that the high glucose-induced activation of the UPP was not an osmotic effect. However, elevated Arkadia expression in the nucleus was significantly decreased by the addition of MG132.

## 4. Discussion

### 4.1. The Role of SnoN and MG132 in an STZ-Induced Diabetic Nephropathy (DN) Rat Model

The mechanism of diabetic nephropathy is multifactorial. The important role of the TGF-*β* signaling pathway in diabetic nephropathy (DN) has been recognized. The transcriptional regulator SnoN plays a fundamental role as a modulator of TGF-*β*-induced signal transduction and subsequent biological responses. Accumulating evidence suggests that SnoN plays a dual role as a corepressor or a coactivator of TGF-*β*-induced transcription [18].

The ubiquitin-proteasome pathway (UPP) is an important nonlysosomal protein degradation pathway. It is able to degrade intracellular proteins efficiently and in a highly selective manner and; in particular, it is able to upregulate or downregulate signaling pathways by degrading the intracellular inhibitor or activating factor of each signaling pathway [18]. Recent studies have shown that SnoN is mainly degraded by the UPP, and regulation of SnoN expression in obstructive nephropathy has also been shown to involve the UPP. Smurf2 is localized in the nucleus and physically associates with SnoN, strongly suggesting that Smurf2 is a ubiquitin E3 ligase that targets nuclear SnoN for proteasome-dependent degradation [19].

However, whether degradation of SnoN is involved in the development of DN is unknown. The most common way to block the UPP is by using small peptides or peptide analogues that bind and inhibit the activity of the 20S core protease, thereby blocking the whole pathway. MG132 is one example of this type of inhibitor; one concern, however, for the use of MG132 is nonspecific proteasome inhibition, and whether global proteasome inhibition has other undesirable effects. In general, effective proteasome inhibition by high doses of MG132 induces apoptotic cell death. By contrast, low doses of MG132 mediate a protective response against oxidative stress [20]. Therefore, MG132 at low daily doses (0.05 mg/kg* in vivo* or 0.5 *μ*mol/L* in vitro*) may predominantly inhibit the elevated proteasomal activity that is caused by diabetes in multiple organs, without inhibiting proteasome activity in normal tissues [21].

This study was therefore carried out in order to clarify the discrepancy in the literature regarding the respective roles played by SnoN and by the UPP in the regulation of diabetic nephropathy (DN). Streptozocin (STZ) is toxic to pancreatic *β* cells and has been widely used to induce diabetes in animal models. The STZ-injected rats exhibited the main characteristics of diabetes mellitus and the changes in the DN markers in our study were similar to those previously reported [22, 23]. Our data support the idea that the transcriptional regulation of SnoN could be considered as a negative regulator of the TGF-*β* signaling pathway in DN as there appears to be a decreased expression of SnoN in STZ-induced renal tissue, with a concomitant increase in TGF-*β* expression. However, upon protease inhibition with MG132, these changes were reduced. The results demonstrated that SnoN was involved in the TGF-*β* signaling pathway in the development of DN.

In this study, we did not examine changes in the mRNA levels of SnoN as a consequence of proteins stimulation by STZ or MG132. This implies that there is no difference in the gene order of SnoN, except for posttranslational modifications, including ubiquitination. In accordance with this, the upregulation of Smurf2 in the kidney is closely correlated with reduction of SnoN after stimulation by STZ. It was also demonstrated that the UPP played a role in activation of the TGF-*β* pathway and induced the progress of DN by ubiquitin degradation of SnoN. Consistent with our observations, Yang et al. identified SnoN as being a negative regulator of TGF-*β* signaling, and SnoN is also utilized as a prognostic marker in estrogen receptor-positive breast carcinomas [24, 25]. Several* in vivo* and* in vitro* studies have provided evidence for the increase in proteasomes in diabetes. For example, exposure of human umbilical vein endothelial cells to high glucose significantly increased the 26S proteasome activity. Proteasomal activity was also increased in skeletal muscle and hearts of STZ-induced diabetic rats and in gastrocnemius muscle of spontaneously diabetic (db/db) mice [26, 27].

The features associated with DN progression are glomerular hypertrophy, thickening of the GBM and mesangial expansion, and eventual loss of glomerular filtration and glomerulosclerosis. Microalbuminuria in diabetic patients predicts the onset of proteinuria, as well as an increased risk of death and cardiovascular events [28]. Our results showed that 24-hour urine protein, urinary albumin-to-creatinine ratios (ACR), and serum BUN levels increased in diabetic control groups; the increasing levels of BUN may indicate progressive renal damage. Recent studies have found that MG132 can protect the kidney against diabetes-induced oxidative damage, inflammation, and fibrosis [29], but the exact pathogenesis has not been completely clarified. Renal fibrosis in DN was induced by the activation of the TGF-*β* signaling pathway, but whether MG132 could treat DN by blocking ubiquitin degradation of SnoN has not been reported. Our research found that, on comparison with the DC group, levels of 24-hour urine protein, ACR, BUN, and collagen content tended to decrease after MG132 intervention. Meanwhile, the pathological changes upon light microscopy observation showed similar trends. This suggests that MG132, by acting as a UPP inhibitor, can protect rat renal tissue from damage, maintain the basement membrane permeability, and reduce urinary protein.

The present study demonstrated that MG132 positively affected these parameters (ACR, BUN, etc.) of DN, but MG132 treatment did not significantly improve blood glucose levels or body weight. Our findings are inconsistent with a previous study that showed a systemic improvement with MG132 when it was used for prevention of diabetes-induced renal pathological changes [30]. We assume that the discrepancy between our study and the previous one is due mainly to the differences of animal models and MG132 administration times [31]. Our results suggest that the therapeutic effect of chronic treatment with MG132 on diabetes-induced renal damage cannot be attributed to systemic improvement, at least not in the Wistar rat diabetic model.

### 4.2. The Role of SnoN and MG132 in Rat Glomerular Mesangial Cells (GMCs) Inducted by High Glucose

SnoN acts as a Smad corepressor by interacting with Smad complexes to inhibit their transcriptional abilities and by recruiting other corepressors and sequestering Smad proteins to prevent their translocation to the nucleus [11]. However, the role of SnoN in rat glomerular mesangial cells (GMCs) stimulated with high glucose is not fully understood. We found that glucose stimulation correlated in a time- and concentration-dependent manner with decreased SnoN expression, but osmotic stress had little effect on the expression of SnoN compared with high glucose. The decrease in SnoN was inhibited following MG132 intervention. These results suggest that high glucose concentration mediates SnoN degradation by means of the UPP and that MG132 may have a positive function in the treatment of DN by inhibiting SnoN ubiquitination, which affects TGF-*β* signaling.

Studies have previously found that Arkadia, as an E3 ubiquitin ligase, associates with SnoN proteins in their free forms, as well as when they are bound to Smad proteins. These findings suggest that Arkadia induces constitutive degradation of SnoN; Arkadia protein expression levels thus appear to determine the intensity of TGF-*β* signaling that is permitted in target cells [15]. However, whether Arkadia enhances TGF-*β* signaling responses in high glucose conditions is unknown. Our experiments show that Arkadia expression was gradually increased in GMCs stimulated with high glucose, compared with the NC group, the effect of which was almost completely reversed by adding MG132. Our results suggest that Arkadia enhances TGF-*β* signaling by inducing degradation of SnoN, which is a negative regulator of TGF-*β* signaling that acts in different ways, and MG132 may have a positive function in the treatment of diabetic nephropathy by inhibiting the disorders involving SnoN ubiquitination.

## 5. Conclusion

In conclusion, the present study has demonstrated that expression of SnoN protein in rats with early DN is downregulated by UPP and that the proteasome inhibitor, MG132, can reduce degradation of SnoN, thus inhibiting activation of the TGF-*β* pathway and conferring a therapeutical effect for DN* in vivo*. SnoN degradation, mediated by Smurf2 or Arkadia, may play an important role in activation of TGF-*β* pathway* in vivo *and* in vitro*. The present results support the hypothesis that ubiquitin degradation of SnoN may be involved in the pathogenesis of DN by specifically activating TGF-*β*/Smad signaling. Components of the ubiquitin-proteasome pathway may be potential therapeutic targets for the treatment of DN.
